# A Distributed Testbed for 5G Scenarios: An Experimental Study

**DOI:** 10.3390/s20010018

**Published:** 2019-12-19

**Authors:** Mohammad Kazem Chamran, Kok-Lim Alvin Yau, Rafidah M. D. Noor, Richard Wong

**Affiliations:** 1 School of Science and Technology, Sunway University, Subang Jaya 47500, Malaysia; mohamma.c@imail.sunway.edu.my (M.K.C.); richardwtk@sunway.edu.my (R.W.); 2 Department of Computer System and Technology, University of Malaya, Kuala Lumpur 50603, Malaysia; fidah@um.edu.my

**Keywords:** D2D communication, 5G, sensor network, sensor, end-to-end delay, USRP, distributed mechanism, Raspberry Pi

## Abstract

This paper demonstrates the use of Universal Software Radio Peripheral (USRP), together with Raspberry Pi3 B+ (RP3) as the brain (or the decision making engine), to develop a distributed wireless network in which nodes can communicate with other nodes independently and make decision autonomously. In other words, each USRP node (i.e., sensor) is embedded with separate processing units (i.e., RP3), which has not been investigated in the literature, so that each node can make independent decisions in a distributed manner. The proposed testbed in this paper is compared with the traditional distributed testbed, which has been widely used in the literature. In the traditional distributed testbed, there is a single processing unit (i.e., a personal computer) that makes decisions in a centralized manner, and each node (i.e., USRP) is connected to the processing unit via a switch. The single processing unit exchanges control messages with nodes via the switch, while the nodes exchange data packets among themselves using a wireless medium in a distributed manner. The main disadvantage of the traditional testbed is that, despite the network being distributed in nature, decisions are made in a centralized manner. Hence, the response delay of the control message exchange is always neglected. The use of such testbed is mainly due to the limited hardware and monetary cost to acquire a separate processing unit for each node. The experiment in our testbed has shown the increase of end-to-end delay and decrease of packet delivery ratio due to software and hardware delays. The observed multihop transmission is performed using device-to-device (D2D) communication, which has been enabled in 5G. Therefore, nodes can either communicate with other nodes via: (a) a direct communication with the base station at the macrocell, which helps to improve network performance; or (b) D2D that improve spectrum efficiency, whereby traffic is offloaded from macrocell to small cells. Our testbed is the first of its kind in this scale, and it uses RP3 as the distributed decision-making engine incorporated into the USRP/GNU radio platform. This work provides an insight to the development of a 5G network.

## 1. Introduction

Fifth generation (5G) is a promising next-generation cellular network armed with new features, particularly device-to-device (D2D) communication that enables direct communication between devices without going through base stations (BSs). This helps to offload traffic from macrocell (MC) BSs to small cell (SC) (i.e., femtocell) BSs, as well as user equipment and devices, including sensors, while increasing network cell coverage via multihop transmission [1,2,3]. In 5G, a node can operate either as a licensed user (or a primary user, PU) to utilize its licensed channels (or cellular channels), or as an unlicensed user (or a secondary user, SU) to explore and utilize white spaces, which are the underutilized licensed channels (or cognitive channels) [4]. D2D enables nodes to access both cellular and cognitive channels to improve spectrum efficiency in order to improve data transmission rate and quality of service (QoS) [5,6,7].

### 1.1. Our Contributions

At present, the majority of the research related to 5G presents theoretical analysis [8,9,10,11,12] and simulation studies [11,12,13,14,15,16]. In general, various theoretical state of the art and open issues are presented In [8], the effects of ultra-densification are investigated In [9], various network architectures, medium access mechanisms, and open issues are presented In [10], as well as routing algorithms to achieve lower interference and a balanced traffic load amoung routes in 5G environment are investigated In [11,12], respectively. In addition, traffic offloading from backbone routes and the central controller to distributed nodes is investigated In [13,14], the transmission delay is predicted based on channel states In [15], and the feasiblity of D2D in 5G environment is investigated In [16]. Some researchers conduct proof of concept experiments; however, the focus is primarily on the physical layer, particularly spectrum management In [2], interference mitigation In [17], channel sensing In [18], as well as on the data link layer, particularly channel hopping (or switches) In [19].

This study focuses on the networking aspect over a 5G-based platform using universal software radio peripheral with GNU radio (USRP/GNU radio) units and Raspberry Pi3 B+ (RP3) processors [20]. GNU radio is an open source software that serves as development toolkit [21] in the platform (for more details see Section 2.3). There are two types of testbeds under investigation in this paper: (a) the traditional testbed comprised of BSs or nodes connected to a single traditional processing unit using a wired medium via a switch [2]; and (b) our distributed testbed in which each BS and node is embedded with a separate processing unit, namely Raspberry Pi3 B+ (RP3). We consider a 5G scenario with: (a) D2D communication; and (b) heterogeneous MC and small cell BSs with different sensing and transmission capabilities, as well as processing capabilities (i.e., using operating systems with different capabilities). Comparison is made on the performance measures of routes via D2D and MC BS. The MC BS selects a route, and informs FC BSs and nodes about the route; subsequently, the FC BSs and nodes setup the route accordingly. Therefore, FC BSs and nodes can be relaxed from performing route selection and channel sensing. Our testbeds are sufficient for the investigation of our contributions, although further extension is suggested in Section 7.

Our contributions are twofold:

- Performance comparison achieved in our distributed testbed based on proof of concept experiments involving multihop transmission, which is necessary in next-generation wireless sensor networks. The BSs and nodes are heterogeneous from MC and SCs with different sensing and transmission capabilities, as well as processing capabilities (i.e., using operating systems with different capabilities).
- Performance analysis of the software and hardware processing delays for communication via D2D and going through BSs over the testbed, which is required for route selection.

### 1.2. Significance of This Paper

There are two main investigations in this paper. Firstly, comparison is made of the performance measures achieved by the traditional testbed and our distributed testbed. Our proposed testbed is distributed in nature and it has a closer resemblance to a real deployed network. The software and hardware processing delays, which are generally ignored in theoretical analysis and simulation, are investigated. Secondly, using the testbeds, comparison is made on the performance measures achieved by: (a) the traditional direct communication with MC BS; and (b) the D2D communication. This comparison is useful for the MC BS to make decision on route selection. This is because, while D2D communication can offload traffic from MC BSs to SC BSs, the end-to-end delay over a multihop transmission increases, and so the direct communication with MC BS can be favorable. The end-to-end delay of a route changes with its operating environment (e.g., the processing capability) and its operation (e.g., the lower read and write rates of RP3 contribute to a higher end-to-end delay and lower packet delivery ratio in our distributed testbed compared to the traditional testbed). Lower end-to-end delay is favorable to support real-time applications integrated with sensors, such as driverless vehicles. To the best of our knowledge, this is the first USRP/GNU radio platform incorporated with RP3 implementation with this scale and functionality.

### 1.3. Organization of This Paper

The rest of this paper is organized as follows. Section 2 presents research background. Section 3 presents related work. Section 4 presents system model and delay measurement. Section 5 presents experimental setup. Section 6 presents experimental results. Section 7 presents our conclusion and future work.

## 2. Background

This section presents an overview of 5G, USRP, GNU radio, and RP3.

### 2.1. 5G

**Figure 1:**
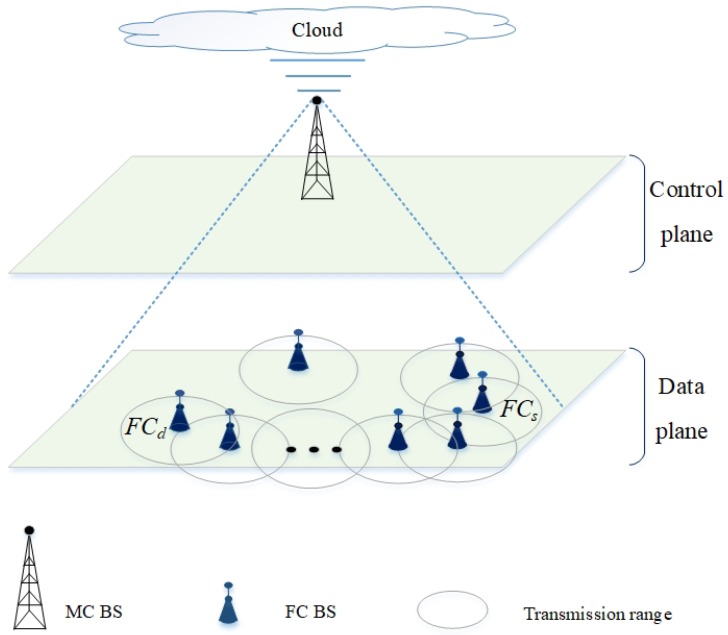
A 5G network that consists of a single MC BS and a number of FC BSs.

The 5G network is a heterogeneous network that consists of different kinds of network cells, including MC and femtocell (FC). The transmission of the BSs and nodes are characterized by different frequency bands and transmission power levels. In Figure 1, a 5G network consists of two main planes: (a) *control plane* consists of MC BSs, which use higher transmission power levels at lower frequency bands, contributing to larger transmission ranges; and (b) *data plane* consists of FC BSs and nodes, which use lower transmission power levels at higher frequency bands, contributing to smaller transmission ranges [22]. The control plane communicates with the cloud, which consists of a central controller (CC) that manages and coordinates global functions, such as route selection. MC BSs can coordinate and communicate among themselves via the cloud [12,23], and this helps them to determine the nodes that each of them must cover. The FC BSs can coordinate and communicate among themselves via D2D if they are within each other’s transmission range, and this helps them to: (a) use a route established from a FC source node $FC_{s}$ to a FC destination node $FC_{d}$ by the CC; and (b) offload traffic from MC BSs. Both MC and FC overlap, and FC BSs can communicate with each other directly. Hence, the MC BS in the control plane can select a route, and inform FC BSs and nodes in the data plane about the route; subsequently, the FC BSs and nodes setup the D2D route accordingly. Therefore, FC BSs and nodes can be relaxed from performing complex tasks, such as route selection.

### 2.2. Universal Software Radio Peripheral

**Figure 2:**
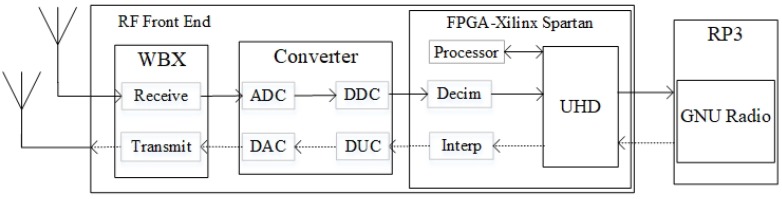
Transmit and receive paths between an antenna and a RP3 via a USRP/GNU radio unit. Solid arrow line is part of a receive path, and dotted arrow line is part of a transmit path.

USRP is an off-the-shelf wireless device that can be configured with a wide range of operating parameters, such as the types of modulation schemes and the channel frequency bands. Our testbed uses USRP N200 series that provides high processing capability. Figure 2 shows a USRP unit that has a set of two omni-directional VERT900 antennas—one for transmission and one for reception—for simultaneous transmissions in two different operating channels within channel frequency bands 850–890 MHz and 2.3–2.4 GHz. The selected channel frequency bands include the television and global system for mobile communication (GSM) bands. The antennas are connected to a daughterboard. There are two types of paths: (a) *receive path* in which analogue signals are received and moved from the radio frequency (RF) front end towards RP3 for reception; and (b) *transmit path* in which digital signals move from RP3 towards the RF front end for transmission.

The USRP consists of three main sections as follows:

- *Wide bandwidth transceive (WBX)* is the RF front end that provides access to different operating channels within a range of 50 MHz of RF bandwidth with 8 bit samples, or 25 MHz of RF bandwidth with 16 bit samples. The maximum transmission power is 100 mW (or 20 dBm) with a noise figure of 5 dB.
- *Converter* consists of: (a) an analogue-to-digital converter (ADC) and a digital down converter (DDC) in the receive path; and (b) a digital-to-analogue converter (DAC) and a digital up converter (DUC) in the transmit path. DDC selects desired signals from an array of signals captured by ADC, while DUC increases the bandwidth of baseband signals so that they are compatible with DAC.
- *Field-programmable gate array (FPGA)*, specifically the Xilinx Spartan 3A-DSP 1800 board [24] used in this platform, consists of: (a) a decimation filter for achieving the required interface bandwidth in the receive path, and an interpolation filter for achieving the opposite in the transmit path; (b) a USRP hardware driver (UHD) block with a software interface that enables various components to communicate among themselves; and (c) a processor block that performs encoding/decoding, modulation/demodulation, timing synchronization, and other signal processes required for software defined radio (SDR) operations. The FPGA communicates with RP3 via power over Ethernet (PoE). It provides connection between: (a) gigabit Ethernet CAT 5E-350 MHz cables, which provide a maximum data rate of 1000 megabits per second (Mbps) connected to a Gigabit switch; and (b) USB3, which provides a maximum data rate of 1600 Mbps. During system initialization, the kernel, which is the fundamental part of an operating system, of GNU radio controls and monitors programs and systems, as well as performs default functions, such as checking and assigning memory space to FPGA [24].

### 2.3. GNU Radio

**Figure 3:**
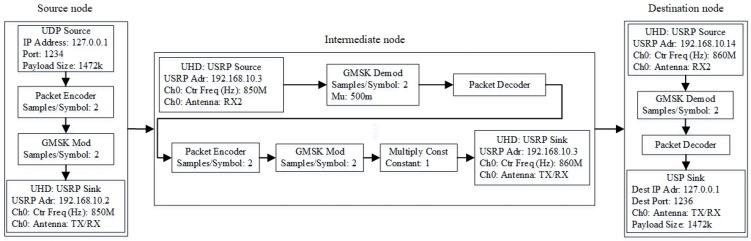
GNU radio flow graph that consists of source, intermediate and destination nodes.

GNU radio, together with its extended version called GNU radio companion (GRC), is an open source SDR that enables users to design: (a) configurable blocks to perform communication tasks using the C++ language; and (b) flow graphs to connect the blocks using the Python language. As shown in Figure 3, the blocks and flow graphs define the roles of the source, intermediate, and destination nodes as follows:

- *Source node*, which is a RP3 unit with an Internet protocol (IP) address (e.g., 192.168.10.2) and a port number (e.g., 1234), generates and sends a data or video stream in the form of frames encapsulated in user datagram protocol (UDP). In GRC, the frames pass through three main components: (a) an *encoder* that converts the frames into packets with a predefined payload length (e.g., 1472 bytes); (b) a *Gaussian minimum shift keying (GMSK) modulator* that converts the packets into modulated signals at baseband (e.g., the minimum non-zero frequencies); and (c) a *USRP sink block* that sets the center frequency (e.g., 850 MHz), channel gain (e.g., 1dB), and sample rate (e.g., 1 MHz). Finally, the signals are broadcasted.
- *Intermediate node* receives signals from a transmitter, which can be a source node or an upstream intermediate node, and transmits them to the next-hop node, which can be a destination or a downstream intermediate node. There are two processes that help to improve the quality of packets before forwarding them in order to reduce interference and address poor channel quality [25]: (a) to demodulate signals to packets, and then to decode packets to frames; and (b) to encode frames to packets, and then to modulate packets to signals. The demodulation and decoding processes are performed at the receiver unit, and then modulation and encoding processes at the transmitter unit.
- *Destination node*, which is a RP3, receives and demodulates signals to packets, and then decodes packets to frames. Then, a UDP sink block sends the frames to an application (e.g., a VLC media player) through a port (e.g., port number 1236 or udp://@:1236).

### 2.4. Raspberry Pi3 B+

Conventionally, a testbed consists of BSs or nodes connected to a single traditional processing unit (e.g., a personal computer or a laptop) using a wired medium via a switch [2] (see Section 5). The BSs and nodes exchange control messages and data packets over the wired and wireless media, respectively. In this paper, each BS and node is embedded with a separate processing unit, namely the RP3 unit. Both control messages and data packets are exchanged over the wireless medium.

There are *three* main advantages. Firstly, *ease of implementation* because nodes can be placed further apart from each other rather than being constrained by physical cables and connections to a single switch. Secondly, *higher cost efficiency (or lower overhead)* because nodes do not communicate with a traditional processing unit. Thirdly, *lower energy consumption* with the use of RP3 compared to personal computers, laptops, and a switch.

However, there are three main disadvantages. Firstly, *lower processing capability*. The RP3 processor (e.g., 1.4 GHz 64-bit quad-core processor with 1 GB non-expandable on-board RAM) is suffice to perform basic tasks and support simple applications (e.g., running GRC in the Linux environment). Secondly, *lower data rate*. The network interface of RP3 has approximately 324 Mbps data rate only, which is low compared to 761 Mbps offered by the gigabit Ethernet of a CORE i7 personal computer. This increases the end-to-end delay of the communication between a RP3 and a USRP. In addition, RP3 is embedded with an SD card, such as a high capacity HC-I class 10 SD card that offers a data rate of 10 megabytes per second (MBps), which is low compared to 550 MBps offered by a solid-state hard drive. This increases the internal delay of the communication between a RP3 and an SD card. This is significant because such communication is commonplace as an operating system (e.g., Ubuntu) is stored as an image in the SD card. It is worth mentioning that the speed (or rate) of read and write on SD cards reduces with the increase of occupied space, and the read rate is generally higher than the write rate as shown in Section 5.3.2. Thirdly, *lower storage space*. The SD card provides low storage space (e.g., 32 GB) for operating systems and software applications.

## 3. Related Work

This section presents related works on testbeds, particularly USRP/GNU radio platforms, for investigating the networking aspect of 5G. It covers two main topics. *Firstly*, the communication delay between nodes along a route. The routes are assumed to be readily available, and they are selected and provided by the central controller. *Secondly*, the testbeds, particularly USRP/GNU radio and RP3 platforms.

### 3.1. Communication Delay between Nodes

In [26], the hardware and software processing delays are investigated on a testbed comprised of ten USRP/GNU radio nodes connected to a single traditional processing unit (i.e., a personal computer) via a switch. The end-to-end delay has shown to reduce since the personal computer can pre-process route selection prior to data transmission.

In [27], the response delay (or the round-trip time) is investigated on a testbed comprised of two USRP/GNU radio nodes connected to a personal computer. The response delay is the duration between the moment the first byte of a packet passes the digital signal processing block of a sender and the moment the first byte of an acknowledgement packet arrives at the sender. The delay is incurred in: (a) the initiation process that includes modulation, sampling, encoding, as well as packet transfer between GNU radio and kernel (or the operating system); (b) the buffering process that collects and stores packets in a buffer (e.g., the buffer of a VLC media player); and (c) the transmission process that receives and sends packets to the FPGA unit of USRP so that they are interpolated before being transmitted via antenna. Measurement shows that the initiation process has the highest time delay, and the transmission process has the lowest time delay.

In [28], the hardware and software processing delays, as well as the response delay, are investigated on a testbed comprised of two USRP/SDR nodes, which serve as the source and destination nodes, connected to a single traditional processing unit (i.e., a personal computer). The source node transmits a data packet to the destination node; and subsequently, the destination node returns a response packet to the source node. The delays are incurred in: (a) processes run in a USRP/SDR node (e.g., operating system and the modulation process); and (b) communication between the two nodes. Measurement shows that the software processing delay incurred in SDR is significantly higher than the hardware processing delay incurred in USRP and the communication delay incurred between the two nodes.

In [29], the response delay, which includes the waiting time of a packet in a queue, is investigated on a testbed comprised of four USRP/GNU radio nodes connected to a single traditional processing unit (i.e., another USRP/GNU radio unit). There are a pair of PU transmitter and receiver, and another pair of SU transmitter and receiver. The SU transmitter must sense the operating channels before transmission so as not to interfere with the PUs. Up to 30% of the delay incurred in the SU transmitter is attributed to channel sensing, which can be reduced to increase throughput at the expense of higher interference level to PUs. Hence, there is a tradeoff between the delay and throughput performances.

In [30], the hardware and software processing delays of different processes are investigated on a testbed comprised of two USRP/GNU radio nodes embedded with separate processing units (i.e., personal computers). The nodes are connected to each other via Ethernet. Examples of the USRP processes are the operating system processes in the kernel, and the decimation filtering in FPGA; and an example of the GNU radio process is the modulation process. During measurement, a 1 μs guard time is included between the processes. A node transmits a ping packet to another node. The packet moves through the transmit and receive paths, and the timestamps for different processes in the USRP/GNU radio node are recorded. Measurement shows that the hardware and software processing delays are highest for processes running in the Kernel. This indicates that the USRP/GNU radio platform has low efficiency providing low network performance, particularly high end-to-end delay.

In this paper, the testbed is comprised of five USRP/GNU radio nodes embedded with separate processing units. Investigation is conducted on multihop transmissions in the network layer.

### 3.2. Testbed of USRP/GNU Radio and Raspberry Pi

This section presents related works on USRP/GNU radio with and without Raspberry Pi.

#### 3.2.1. USRP/GNU Radio without Raspberry Pi

**Figure 4:**
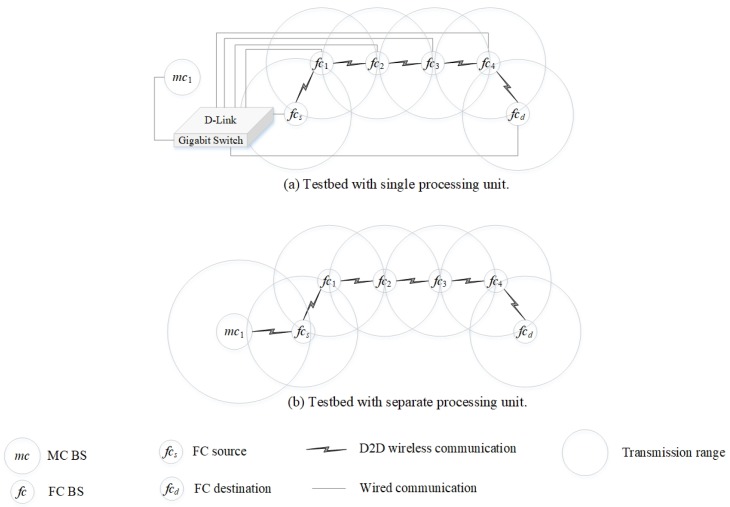
Two scenarios in experimental setup.

In [2,31,32,33,34,35,36], a testbed consists of BSs or nodes connected to a single traditional processing unit (e.g., a personal computer or a laptop) via a switch (i.e., Gigabit D-link) [2] (see Figure 4a). This allows the central controller to exchange control messages with BSs and nodes via a switch in a centralized manner, while the BSs and nodes can exchange data packets using the wireless medium [37] in a distributed manner. Examples of control messages include those that carry information about channel sensing and selection, route discovery and selection, and handshaking (e.g., request, acknowledgement, and response messages). Therefore, the response delay of a D2D type of communication in a real testbed is a cause of concern because of the sensitivity of wireless communication and the delay incurred due to the distance between a node pair. The response delay is important in D2D communication because if it may not fulfill the delay requirement (or higher than a threshold), MC BS must be used. This paper focuses on the response delay, which is end-to-end in nature, between a source node from first transmitted packet up to last received one.

In this paper, each BS or node is embedded with a separate processing unit, particularly RP3 as the core processing unit, to provide a more realistic wireless testbed, and so a single traditional processing unit is not needed.

#### 3.2.2. USRP/GNU Radio with Raspberry Pi

In [38], a single USRP/GNU radio embedded with Raspberry Pi3 is used to generate signals in the range of FM radio frequency bands. In addition, a radio station also generates signals in the frequency bands. Subsequently, the signals generated by the Raspberry Pi3 and radio station are measured using a spectrum analyzer, and a comparison is made. The quality of signals generated by the USRP/GNU radio, despite using a lower transmission power at lower frequency bands, has shown to be close to that from a radio station. In general, the received signals from USRP/GNU radio has a lower throughput and energy consumption.

In [39], a single USRP/GNU radio node embedded with Raspberry Pi3 is used to perform energy-based channel sensing in order to detect PUs activities. There are two main sources of energy consumption in Raspberry Pi3: (a) software initiation; and (b) the calculation of the available memory of the kernel running GNU radio libraries. Channel sensing has shown to incur the highest delay. Similar investigation is performed In [20]. Energy consumption in Raspberry Pi3 has shown to be considerably lower compared to that in personal computer.

In [40], a single register transfer level (RTL) dongle embedded with a Raspberry Pi transmits and receives in the frequency bands 24–1850 MHz. The general-purpose input/output (GPIO) pins of the Raspberry Pi is used to generate and transmit pulse width modulation (PWM). The energy consumption of the RTL dongle embedded with Raspberry Pi has shown to be less than 3 watts, and so the dongle and Raspberry Pi can be powered by portable batteries. In addition, the use of Raspberry Pi has shown to enable the detection of a wide range of frequency bands while incurring low energy consumption.

In [41], a single USRP/GNU radio node connected to a personal computer, which serves as the transmitter, broadcasts signals to a RTL dongle embedded with a Raspberry Pi, which serves as the receiver. The testbed consists of a low-cost radio community that transmit at two frequency bands, namely 915 MHz (or the ISM band) and 40.68 MHz (or the FM radio frequency band). The testbed has demonstrated the capability of Raspberry Pi for transmitting and receiving signals in these frequency bands, and the quality of reception depends on the transmission power and the height of the antenna of the transmitter.

In [42], a testbed consists of two USRP/GNU radio nodes: (a) a static node, which is connected to a personal computer, serves as the ground BS; and (b) a dynamic node, which is embedded with Raspberry Pi3, is installed on an unmanned aerial vehicle (or a drone). The ground BS receives location information from the drones so that it can monitor the location of the drone. The ground BS and drone exchange messages in the frequency bands 400–4400 MHz. The testbed has demonstrated the capability of Raspberry Pi3 for setting up communication and processing information with lower energy consumption.

In [43], a testbed consists of three main USRP/GNU radio nodes: (a) a SU source node, which is connected to a personal computer; (b) a SU intermediate node, which is embedded with Raspberry Pi3, that performs energy-based channel sensing; and (c) a SU destination node, which is embedded with Raspberry Pi3. The rest of the nodes are PUs. The SU source node transmits data packets to the SU destination node in multiple hops without interfering with the random PUs’ activities. The channel sensing delay incurred by the SU intermediate node embedded with Raspberry Pi3 has shown to be twice of that incurred by the SU source node connected to a personal computer.

In this paper, our testbed consists of five USRP/GNU radio nodes embedded with Raspberry Pi3 B+ (RP3) that constitutes a source node and four intermediate nodes. In addition, a personal computer serves as the destination node. The USRP/GNU radio performs communication, and the RP3 performs processes. While existing works in the literature [20,38,39,40,41,42,43], focus on the capability and compatibility of USRP/GNU radio and RP3, this paper focuses on end-to-end hardware and software processing delays between a source node and a destination node, and the use of the delay measurement for route selection (i.e., either via D2D or MC BS).

## 4. System Model and Delay Measurement

This study measures network performance, particularly end-to-end delay, packet delivery ratio, and throughput, under different scenarios characterized by the characteristics of 5G, and compares results obtained from two types of testbeds, namely: (a) a testbed with a single traditional processing unit via a switch (see Figure 4a), (see Section 5.3.1); and (b) a testbed with separate processing unit (see Figure 4b), particularly RP3, embedded in each node and BS without using a switch (see Section 5.3.2).

The rest of this section presents system model in Section 4.1, D2D link delay in Section 4.2.1, and D2D end-to-end delay in Section 4.2.2.

### 4.1. System Model

**Figure 5:**
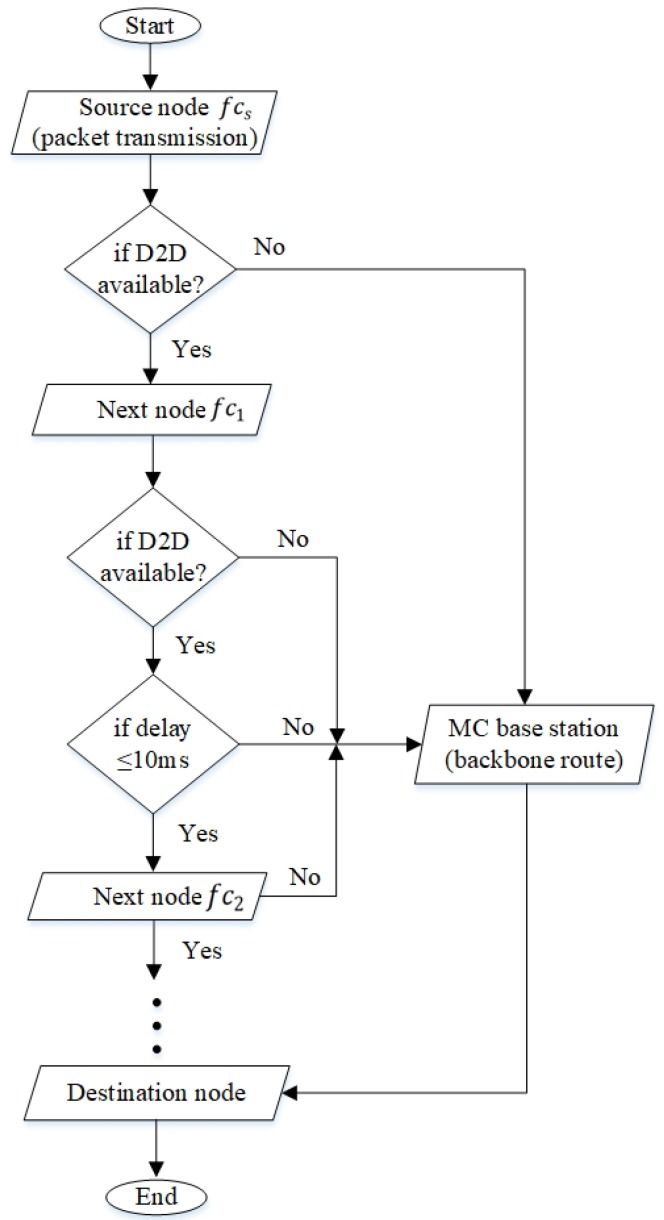
Conditions for selecting a primary route (via D2D).

The system topology consists of femtocell nodes $fc_{f}\in \left\{fc_{1},fc_{2},\ldots ,fc_{\left|F\right|}\right\}$ located within the transmission range of a MC BS. Nodes can transmit UDP packets in one of the routes in a route set, specifically $k_{k}\in K=\left\{k_{1},k_{2},\ldots ,k_{\left|K\right|}\right\}$. Each link $l_{n}\in L=\left\{l_{1},l_{2},\ldots ,l_{\left|L\right|}\right\}$ uses one of the channels $c_{c}\in C=\left\{c_{1},c_{2},\ldots ,c_{\left|C\right|}\right\}$. Each route $k_{k}=\cup \;l_{n}\in L$ consists of a set of links from a femtocell source node to a femtocell destination node. a femtocell source node $fc_{s}$ sends packets to a femtocell destination node $fc_{d}$ along a primary route $k_{k}=\left(fc_{s},fc_{1},fc_{2},fc_{3},fc_{4},fc_{d}\right)$, which is D2D and multihop in nature. In a testbed with a single traditional processing unit, the femtocell nodes $fc_{s},fc_{1},fc_{2},fc_{3},fc_{4},fc_{d}$ are connected to a MC BS, which serves as the centralized processor, via a switch as shown in Figure 4a (see Section 5.3.1 for more descriptions). On the other hand, in a testbed with separate processing units, each femtocell node is embedded with a separate processing unit, namely RP3, as shown in Figure 4b (see Section 5.3.2 for more descriptions). In this paper, a primary route has up to five hops. The primary route uses cognitive channels (or white spaces in licensed channels), and the secondary route uses cellular channels (or the licensed channels). The use of primary routes helps to reduce the congestion level of MC BS [4]. However, when the primary route becomes unavailable or broken, then a secondary route $k_{k}=\left(fc_{s},mc_{1},fc_{d}\right)$, which passes through the macrocell BS $mc_{1}$. The route selection between primary and secondary routes is shown in the form of a flowchart in Figure 5 and an algorithm in Algorithm 1.
**Algorithm 1** Route selection between the primary route (via D2D) and the secondary route (via MC BS)1:**procedure**Route selection2: **for** $k_{1}=\left(fc_{s},fc_{1},fc_{2},fc_{3},fc_{4},fc_{d}\right)$ and $k_{2}=\left(fc_{s},mc_{1},fc_{d}\right)$ **do**3:  **if** route $k_{1}$ is available **then**4:   $fc_{s}$ send packet to $fc_{1}$5:   **if** $fc_{1}$ is not available **then**6:    packet goes through $mc_{1}$ (secondary route) to $fc_{d}$7:   **end if**8:   **if** $fc_{2}$ is available **then**9:    check the second condition:10:    **if** $t^{fc_{1}tofc_{2}}$ is ≤α **then**11:     $fc_{1}$ send packet to $fc_{2}$12:    **end if**13:   **end if**14:   **if** $fc_{2}$ is not available **then**15:    packet goes through $mc_{1}$ to $fc_{d}$16:   **end if**17:   **if** $fc_{3}$ is available **then**18:    check the second condition:19:    **if** $t^{fc_{2}tofc_{3}}$ is ≤α **then**20:     $fc_{2}$ send packet to $fc_{3}$21:    **end if**22:   **end if**23:   **if** $fc_{3}$ is not available **then**24:    packet goes through $mc_{1}$ to $fc_{d}$25:   **end if**26:   **if** $fc_{4}$ is available **then**27:    check the second condition:28:    **if** $t^{fc_{3}tofc_{4}}$ is ≤α **then**29:     $fc_{3}$ send the packet to $fc_{4}$30:     $fc_{4}$ send the packet to destination $fc_{d}$31:    **else** packet goes through $mc_{1}$ to $fc_{d}$32:    **end if**33:   **end if**34:  **end if**35: **end for**36:**end procedure**

The end-to-end delay $t^{k_{k}}$ of a primary route $k_{k}$ increases with the number of hops [44], and it must be less than a threshold $t^{k_{k}}<\alpha$, where α=10 ms is imposed by the IEEE 802.15.4 standard [14,45]. The secondary route is selected if the threshold is not fulfilled. The threshold α is imposed due to the need to reduce end-to-end delay in order to support and deploy real-time applications, including applications integrated with sensors such as driverless vehicles, in 5G. Long software and hardware processing delays can increase the queue size of base stations and nodes, and so they affect network performance, such as reducing packet delivery ratio [46,47].

In this paper, route selection is made by a central controller, and so the underlying routes, as well as the channels of the links in the routes, are readily available. There is a single MC BS that selects a route, and informs FC BSs and nodes about the route; subsequently, the FC BSs and nodes setup the route accordingly. Further extension to the testbed, such as increasing the number of MC BSs, is suggested in Section 7. The investigation takes into account the effects of the characteristics of 5G, including heterogeneity that involves nodes with different features and characteristics (i.e., different transmission power, frequency range, and strength of operating system).

### 4.2. Delay Measurement

This section presents D2D link and end-to-end delay, respectively.

#### 4.2.1. D2D Link Delay

**Figure 6:**
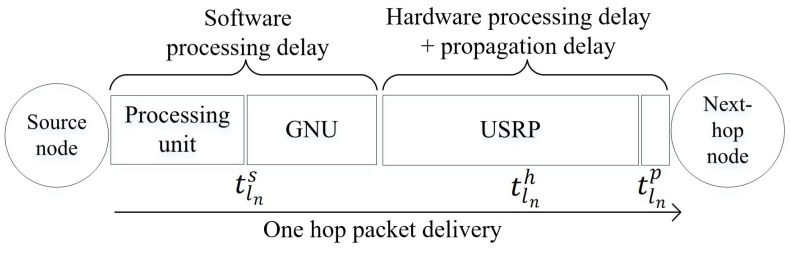
D2D link delay for a single-hop transmission over a link $l_{n}$. The processing unit can be either a personal computer or a RP3.

The D2D link delay (or per-hop delay) consists of three kinds of delays as shown in Figure 6. Firstly, the *software processing delay* $t_{l_{n}}^{s}$ is incurred in GNU radio running on processing unit, such as personal computer and RP3, to process packets, such as encoding and modulating packets (see Section 2.3), over a link $l_{n}$. Secondly, *hardware processing delay* $t_{l_{n}}^{h}$ is incurred in USRP to convert electrical packets to RF signals for transmission in the transmit path, and to convert RF signals to electrical packets upon reception in the receive path, over a link $l_{n}$. Thirdly, the propagation delay $t_{l_{n}}^{p}$ is incurred for the RF signal to travel from one USRP/GNU radio node to another over a link $l_{n}$; however, it is negligible compared to software and hardware processing delays [27].

The D2D link or per-hop delay $t_{l_{n}}$ for link $l_{n}\in L$ is as follows:

(1) $$t_{l_{n}}=t_{l_{n}}^{s}+t_{l_{n}}^{h}$$

#### 4.2.2. D2D End-to-End Delay

The D2D end-to-end delay $t^{k_{k}}$ of a route $k_{k}\in K=\left\{k_{1},k_{2},\ldots ,k_{\left|K\right|}\right\}$ is the accumulation of the D2D link delay at each link, which consists of software and hardware processing delays, as follows:

(2) $$t^{k_{k}}=\sum_{l_{n}\in k_{k}}t_{l_{n}}$$

## 5. Experimental Setup

This experiment investigates the link (or per hop) and end-to-end delays of a route via D2D communication among heterogeneous BSs and nodes (i.e., MC and FCs) (see Section 4 for more details).

### 5.1. Experiment Parameters

The experiment uses: (a) licensed channels, including the TV frequency bands (i.e., 850–890 MHz) and long term evolution (LTE) frequency bands (i.e., 2.3–2.4 GHz) [48]; and (b) unlicensed channels, particularly the industrial, scientific and medical (ISM) frequency bands (i.e., 2.4 GHz). The experimental parameters are summarized in Table 1. In addition, the USRP parameters are presented in Section 2.2, and the GNU radio parameters and flow graph are presented in Section 2.3 and Figure 3, respectively. In GNU radio, the bandwidth can be represented by sample rate (or the number of samples per second). The transmission power used for the range of frequency bands within 850 and 890 MHz with a 1 dB set gain is 10 mW for 10 dBm.

### 5.2. Experiment Measurement

The software and hardware processing delays are measured using Wireshark [49], which is an open source packet analyzer (or a packet sniffer) software. For each packet transmission, Wireshark is used to measure the delays incurred by the processing unit (i.e., the time period for a media stream to be transformed into packets), the USRP (i.e., the time period for a packet to traverse from the USRP sink block to the antenna for transmission), and the propagation from one USRP unit to another. The IP address of the source node of the packet can be identified using Wireshark. The delay incurred by GNU radio (i.e., time period for a packet to traverse from the USRP source block to the USRP sink block) can be measured using Python, whereby the delay is given by the time difference between the USRP source block and the USRP sink block.

Meanwhile, there are *two* types of delays that are negligible: (a) the delay incurred for the initialization of different software prior to converting frames into packets; and (b) the delay incurred for signal propagation because the same transmit and receive components are used in both PCU and RPU.

### 5.3. Experiment Testbeds

**Figure 7:**
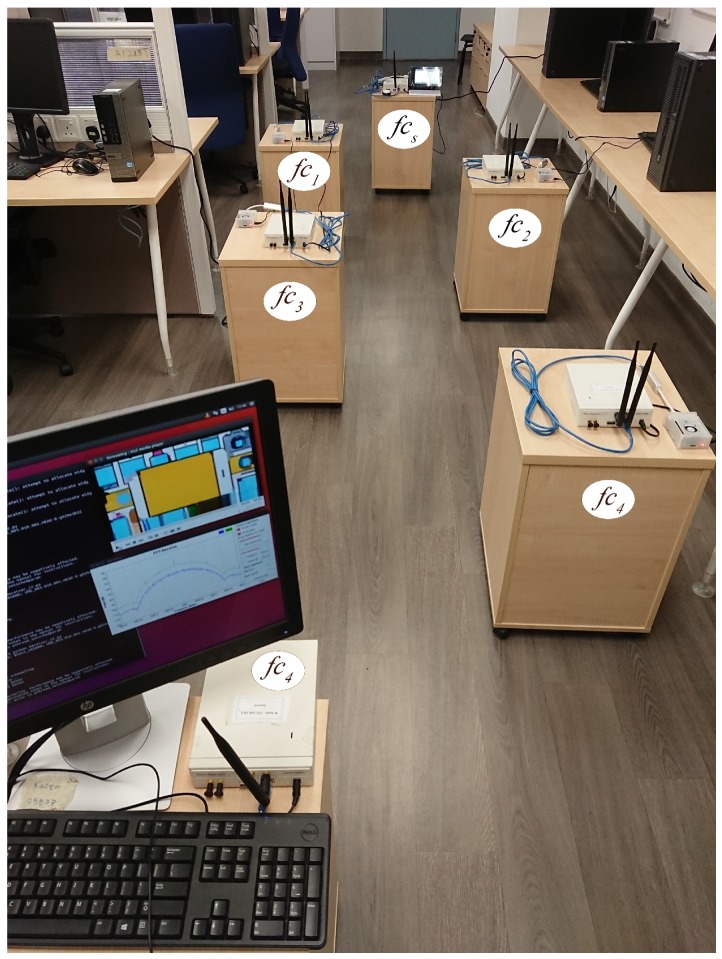
An experimental setup for a D2D route with five hops using RP3 in the RPU testbed, which is equivalent to Figure 4b.

The testbed has a route with five hops as shown in Figure 7. A video stream in the form of UDP packets is generated at a source node, forwarded along a route with four intermediate nodes, and received at a destination node. Video stream is chosen in this experiment due to its stringent QoS compared to data packets. Two types of testbeds are considered. Figure 4a shows a testbed with a single processing unit (PCU), in which a number of heterogeneous MC and FC BSs connect to a single traditional processing unit (i.e., a personal computer) using a wired medium via a switch. Figure 4b shows a testbed with separate processing units (RPU), in which the MC and FC BSs are embedded with separate processing units, namely RP3, and the FC BSs are located within the MC BS proximity. The rest of this section presents the two types of testbeds and their differences.

#### 5.3.1. Testbed with a Single Processing Unit (PCU)

**Figure 8:**
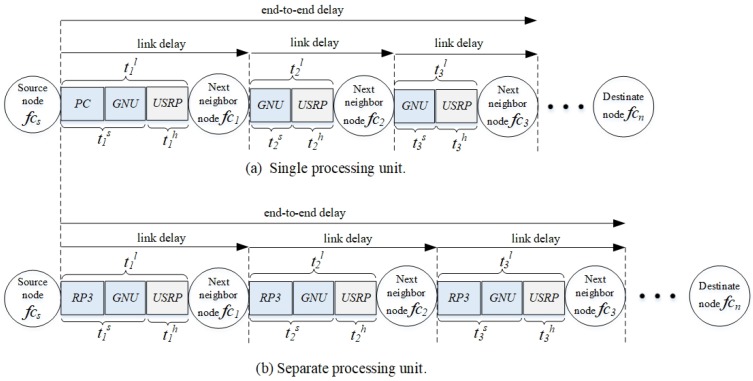
The end-to-end delay of a route in the two testbeds. The delays are shown in same sized blocks although the time period of each block may be different.

Figure 8a shows a testbed in which BSs or nodes are connected to a single traditional processing unit (PCU) (i.e., a personal computer) via a switch [2]. The personal computer has a CORE i7 processor, a 8 GB RAM, and a 1 terabyte of storage space in hard disk, and it provides a centralized approach to handle D2D communication. In other words, a single GNU radio flow graph (see Figure 3 in Section 2.3) is installed in the personal computer. The BSs and nodes exchange control messages with the personal computer via Gigabit Ethernet CAT 5E-350 MHz cables connected to a Gigabit switch, and data messages among themselves via wireless transmission. The control message has a packet size of 826 megabytes and a payload size of 1472 bytes, and it contains the source node IP address, destination node IP address, available channels, acknowledgement, packet types, and timestamp. The source node incurs *three* types of delays: (a) the software processing delay incurred in the personal computer (i.e., to generate and segment video stream into UDP packets); (b) the software processing delay incurred in GNU radio (see Section 2.3 for the processes); and (c) the hardware processing delay incurred in USRP (see Section 2.2 for the processes) and the propagation delay in the transmission incurred from the current node to the next-hop node. Subsequently, the intermediate nodes do not incur the software processing delay in the personal computer. Based on Equation (2), the end-to-end delay of a route $k_{k}\in K$ is as follows:

(3) $$\begin{array}{cc} t^{k_{k}} & =\left(t_{l_{1}}^{PC}+t_{l_{1}}^{GNU}+t_{l_{1}}^{h}\right)+\left(t_{l_{2}}^{GNU}+t_{l_{2}}^{h}\right)+\ldots +\left(t_{l_{n}}^{GNU}+t_{l_{n}}^{h}\right) \end{array}$$

where $t_{l_{*}}^{PC}$ is the single processing unit delay, $t_{l_{*}}^{GNU}$ is the GNU radio processing delay, and $t_{l_{*}}^{h}$ is the hardware processing delay (see Figure 6). The software processing delay is $t_{l_{n}=l_{1}}^{s}=t_{l_{1}}^{PC}+t_{l_{1}}^{GNU}$ for the first hop and $t_{l_{n}\neq l_{1}}^{s}=t_{l_{*}}^{GNU}$ for the subsequent hops. The $t_{l_{*}}^{GNU}$ includes the response time incurred for a node of a route to request for next-hop node information (e.g., the next-hop node IP address and operating channel) from the personal computer.

#### 5.3.2. Testbed with Separate Processing Units (RPU)

Figure 8b shows a testbed in which each BS or node is connected to a separate processing unit (RPU) (i.e., a RP3), and so a switch is not required. RP3 provides a distributed approach to handle D2D communication. In the route, $fc_{s}$ transmits to next-hop $fc_{1}$ in 850 MHz. Then, $fc_{1}$ receives in 850 MHz and transmits in 860 MHz; $fc_{2}$ receives in 860 MHz and transmits in 870 MHz; $fc_{3}$ receives in 870 MHz and transmits in 880 MHz; $fc_{4}$ receives in 880 MHz and transmits in 890 MHz to the destination node $fc_{d}$.

The BSs and nodes exchange control and data messages among themselves via wireless transmission. Each node incurs three types of delays: (a) the software processing delay incurred in RP3; (b) the software processing delay incurred in GNU radio; and (c) the hardware processing delay incurred in USRP and the propagation delay. In contrast to the testbed with a PCU (see Section 5.3.1), the intermediate nodes incur the software processing delay in the RP3. Unlike PCU, in RPU, every single node (either source, intermediate, or destination node) receives decision on the next-hop node and the transmission channel, which incurs $t_{l_{n}}^{RP3}$ (see Figure 8), from the personal computer. The node can also receive such information from the personal computer. Since RP3 has limited processing capability, the software processing delay for the software processes is non-negligible at each node in RPU, causing longer end-to-end delay compared to that in PCU. Based on Equation (2), the end-to-end delay of a route $k_{k}\in K$ is as follows:

(4) $$t^{k_{k}}=\sum \left(t_{l_{n}}^{RP3}+t_{l_{n}}^{GNU}+t_{l_{n}}^{h}\right)$$

where the software processing delay is $t_{l_{*}}^{s}=t_{l_{*}}^{RP3}+t_{l_{*}}^{GNU}$ for all hops.

**Figure 9:**
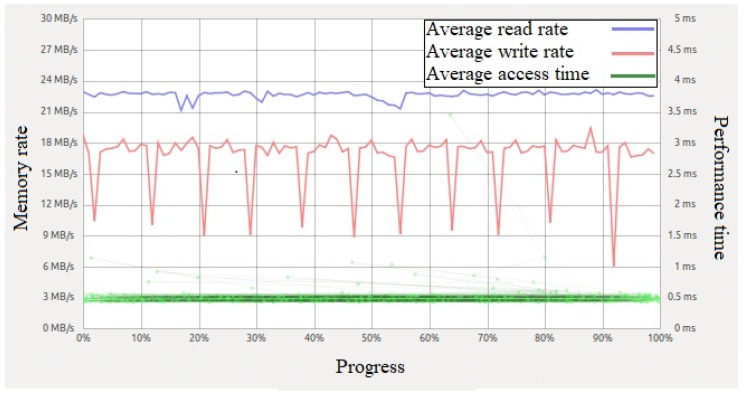
Comparison between read and write performance of SD card on RP3.

The software processing delay for personal computer $t_{l_{*}}^{PC}$ and RP3 $t_{i_{*}}^{RP3}$ are different. There are two types of access rates: (a) the *read rate* is the speed in which a node reads information and reading is needed during transmission; and (b) the *write rate* is the speed in which a node writes information and writing is needed during reception. For both read and write, the access time defines their rates. Access time is the average duration for the kernel to access a partition and perform a task, including read and write. In this evaluation, the kernel performs read and write operations from the boot partition of the Linux Ubuntu MATE operating system installed in RP3. The read rate is considered in a source node, both read and write rates are considered in an intermediate node, and the write rate is considered in a destination node. In general, the average read rate is higher than the average write rate. In personal computer, the read and write rates are much higher; specifically the hard disk drive (HDD) of a personal computer has a drives spin of 7200 revolutions per minute (RPM) in our testbed, and the read and write rates are approximately 80 MBps and 50 MBps, respectively. In RP3, the read and write rates are approximately 22.6 MBps and 16.6 MBps, respectively, for the memory card (i.e., the SD card) on RP3. Due to the lower write rate, the packet queue size at intermediate and destination nodes increase with the number of packets. Hence, RP3 has a limited performance. Figure 9 shows the average read and write rates for reading and writing 1000 samples from/to the boot partition of RP3 with an average access time of 0.48 ms. The average read rate hovers between 22 and 23 MBps, and the average write rate varies between 9 and 20 MBps. The average access time refers to the duration of reading a packet from or writing a packet to the boot partition, and it has an average value of 0.56 ms. The significant lower read and write rates of RP3 can contribute to a higher end-to-end delay and lower packet delivery ratio in RPU compared to PCU.

For an intermediate node, it receives and processes (i.e., decodes and demodulates) packets before it writes them in its memory, then it reads them from the memory and processes (i.e., encodes and modulates) the packets for transmission. The intermediate node must write an entire packet before it can read the packet again for transmission, which is a phenomenon called buffering that occurs during initiation. This allows packets to be fully converted (or digitized) before transmission; however, it causes a higher end-to-end delay as the number of hops increases.

## 6. Experimental Results

This section compares the PCU and RPU performance measures in Section 6.1, and the packet delivery via primary route (i.e., via D2D) and secondary route (i.e., via MC BS) in Section 6.2.

### 6.1. Performance Comparison between PCU and RPU

**Figure 10:**
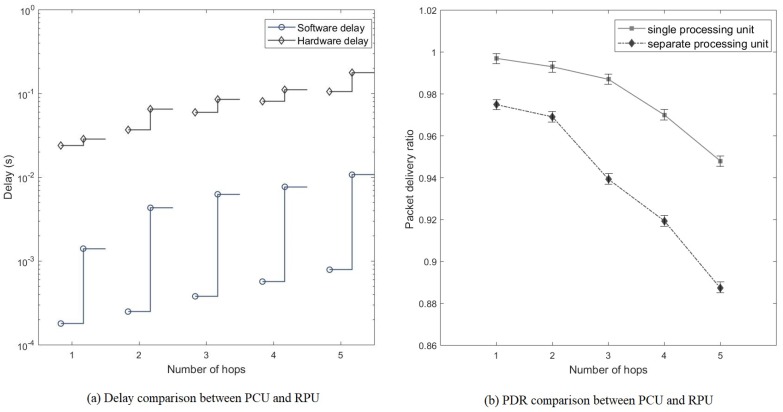
End-to-end delay and packet delivery ratio for PCU and RPU. (**a**) Comparison of software and hardware processing delays between PCU and RPU. (**b**) Comparison of packet delivery ratio between PCU and RPU.

Figure 10a presents a logarithmic graph that shows the total delay, as well as the software and hardware processing delays, for a route from the source node to the destination node. For each number of hops, the results are presented using a pair of bars: the left represents PCU, and the right represents RPU. The total delay is higher in RPU, and it increases as the number of hops increases, specifically the total delay increases from 0.024102s for a single hop to 0.10566s for five hops in PCU, and from 0.02992 s for a single hop to 0.18924s for five hops in RPU. Compared to the hardware processing delay, the software processing delay is significantly lower due to the high processing capability of the personal computer in PCU; specifically, the software processing delay is 0.00018s (or 0.7468%) for a single hop, 0.00023s (or 0.6189%) for two hops, 0.00038s (or 0.6316%) for three hops, 0.00057s (or 0.6958%) for four hops, and 0.00079s (or 0.7476%) for five hops. However, the software processing delay is significantly higher than that in PCU (i.e., approximately five times higher); specifically, the software processing delay is 0.00141s (or 4.7125%) for a single hop, 0.00433 s (or 6.252%) for two hops, 0.00628s (or 6.8484%) for three hops, 0.00771s (or 6.5844%) for four hops, and 0.01074s (or 5.675%) for five hops. This is because, in RPU, RP3 has a lower processing capability, causing a higher total delay in each hop.

Figure 10b shows that the packet delivery ratio reduces as the number of hops in a route from the source node to the destination node increases because more intermediate nodes are affected by the ambient noise in the operating environment. Specifically, the packet delivery ratio reduces from 99.7% for a single hop to 94.8% for five hops in PCU, and it reduces from 97.49% for a single hop to 88.73% for five hops in RPU.

### 6.2. Comparison of Packet Delivery via Primary and Secondary Routes

**Figure 11:**
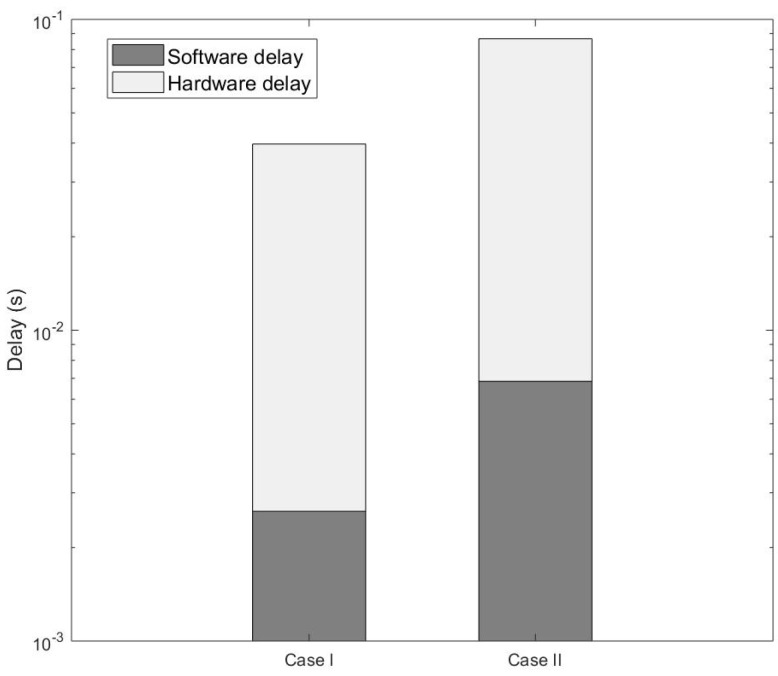
Comparison of software and hardware processing delays between Case I and Case II. In Case I, a two-hop communication is performed via MC BS in PCU. In Case II, a two-hop communication is performed via D2D in RPU.

This section compares the performance of a communication that uses: (a) a multihop primary route (via D2D); and (b) a secondary route (via MC BS). This is because a source node can communicate with a destination node via either a multihop D2D route or going through a BS. Nevertheless, the delay in a multihop D2D route must be less than 10 ms (see Section 4). Figure 11 shows that a packet delivered via a D2D route has a higher end-to-end delay than its counterpart route using MC BS.

**Figure 12:**
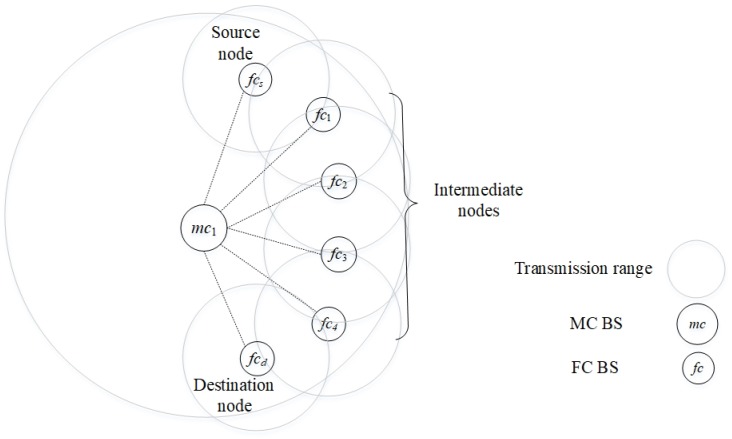
A D2D route from a FC source node $fc_{s}$ to a FC destination node $fc_{d}$.

**Figure 13:**
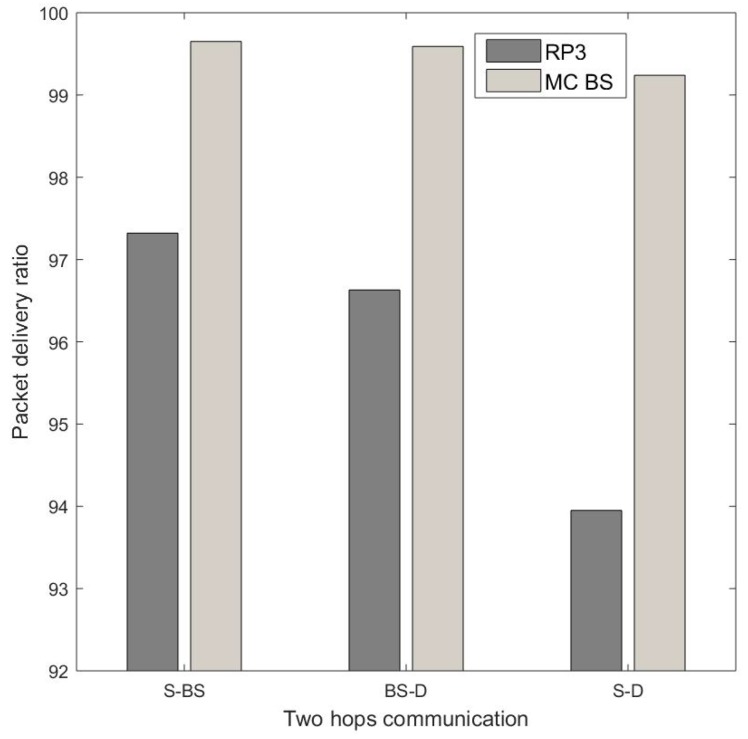
Comparison of the two-hop packet delivery via D2D and MC BS.

This section investigates the end-to-end delay of a primary route when nodes are embedded with RP3, and compares it with that of a secondary route, in which only source and destination nodes are embedded with RP3, and the BS is implemented using a personal computer. In Figure 12, a RP3 source node $fc_{s}$ can communicate with a RP3 destination node $fc_{d}$ via either: (a) Case I which is a direct communication with MC BS $MC_{1}$ (i.e., a personal computer), specifically $fc_{s}-MC_{1}-fc_{d}$ in PCU; or (b) Case II which is a two-hop route using D2D communication, specifically $fc_{s}-fc_{1}-fc_{d}$ in RPU, whereby $fc_{1}$ is an intermediate node embedded with RP3. The total delay of Cases I and II are 0.039736 s and 0.08666 s, respectively, and so the total delay of Case II is more than twice higher than that in Case I. In RPU, the total software and hardware processing delay of a D2D communication from the source node up to the intermediate node is 0.03384s; while in PCU, the total software and hardware processing delay from the source node up to the MC BS is 0.01655 s. In RPU, the total software and hardware processing delay from the intermediate node $fc_{1}$ to the destination node is 0.05282 s; while in PCU, the total software and hardware processing delay from the MC BS to the destination is 0.02318 s. Hence, Case I has a lower total delay as compared to Case II due to its greater processing capability. Figure 11 shows the total delay, which includes software and hardware processing delays, of a direct communication with MC BS and a multihop D2D route. Figure 13 shows the packet delivery ratio via D2D and MC BS. A source node transmits a packet towards a destination node, the packet goes through intermediate node as the destination node is beyond the transmission range of the transmitter. For this packet transmission, the intermediate node is first selected (i.e., Case I), and then the MC BS is selected (i.e., Case II). From the source node to the intermediate node in Figure 13, the MC BS has a 99.65% packet delivery ratio, while the node with RP3 has 97.32%. From the intermediate node to the destination node, MC BS delivers 99.59% of the packets, while the node with RP3 delivers 96.63% of the packets. Therefore, in Figure 13 the total packet delivery ratio from a source node to a destination node via MC BS is 99.24%. However, the packet delivery ratio from the source node to the destination node via RP3 is 93.95%, which is about 7% lesser.

**Figure 14:**
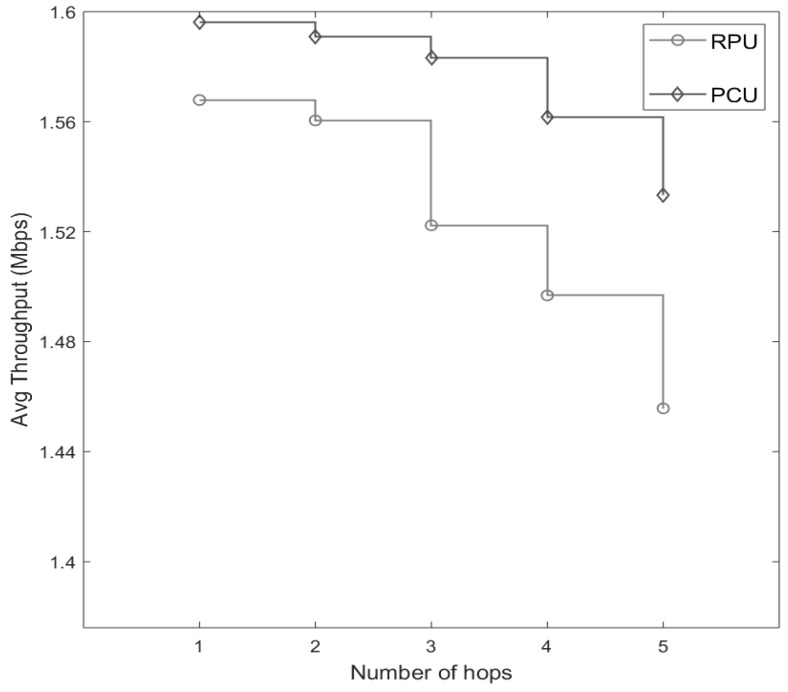
Throughput comparison between RPU and PCU.

In Figure 14, a throughput comparison is made between PCU and RPU. Higher throughput refers indicates a higher successful packets transmission rate [26]. Throughput reduces as the number of hops increases for both PCU and RPU; however, PCU achieves a higher throughput compared to RPU because of higher packet delivery ratio (see Figure 10b).

## 7. Conclusions and Future Work

This paper presented an experimental study to compare performance measures in a testbed with a single processing unit (PCU) and a testbed with separate processing units (RPU). The testbed consists of nodes implemented using universal software radio peripheral with GNU radio. In PCU, base stations and user equipment, including sensors, connect to a single centralized traditional processing unit (e.g., a personal computer or a laptop) via physical cables and a switch. On the other hand, in RPU, each BS or node is embedded with a separate processing unit, particularly Raspberry Pi3 B+. While PCU is a widely used testbed in the literature, nodes are constrained to be located at close proximity to the centralized processing unit. Meanwhile, RPU has a closer resemblance to a real deployed network, and it has not been investigated in the literature, and so it is the focus of this paper. Our experimental results showed that: (a) the end-to-end delay is lower in PCU as control messages are exchanged via a switch using gigabit Ethernet; and (b) the per-hop and end-to-end delays increase with the number of hops in RPU. However, in RPU, device-to-device communication between nodes from a source node to a destination node can offload traffic from BS, which is one of the promising features of 5G. Therefore, this paper presents a case study in which the intermediate node of a two-hop route can be: (a) a node (via D2D); or (b) a macrocell BS. While the preceding case can reduce the traffic amount at a macrocell BS, it can increase end-to-end delay and reduce packet delivery ratio compared to the latter case due to its lower processing capability.

As for future work, we aim to relax the assumptions made in this article to enable a macrocell base station (MC BS) to receive updates from femtocell base stations and nodes. Examples of such updates are the packet delivery ratio and per-hop delay, which allows MC BS to make decision on route selection based on the updates under unpredictable and dynamic operating environment.

## Figures and Tables

**Table 1:** Experimental parameters.

Category	Parameter	PCU	RPU
Experiment	Duration	300 s	300 s
USRP	Number of channels	6	6
	Transport layer	UDP	UDP
	Bandwidth	1.6 Mbps	1.6 Mbps
	Transmission power	10 dBm	10 dBm
Antenna	Carrier frequency	850 MHz	850 MHz
Computer	Operating system	Ubuntu	-
Switch	Number of units	1	-
	Number of inputs	6	-
RP3	Operating system	-	Ubuntu-Mate
PoE	Number of units	-	5
	Number of inputs	-	1

## Abbreviations

The following abbreviations are used in this manuscript:

USRP	Universal software radio partnership
RP3	Raspberry Pi3 B+
5G	Fifth generation
D2D	Device to device
MC	Macrocell
FC	Femtocell
PC	Picocell
CC	Central controller
BC	Base station
WBX	Wide bandwidth transceiver
FPGA	Field-programmable gate array
RF	Radio frequency
ADC	Analogue-to-digital converter
DAC	Digital-to-analogue converter
DUD	Digital up converter
DDC	Digital down converter
UHD	USRP hardware driver
SDR	Software defined radio
PoE	Power over Ethernet
Mbps	Megabits per second
USP	Universal serial bus
CAT	Category
GRC	GNU radio companion
IP	Internet protocol
UDP	User datagram protocol
GMSK	Gausian minimum shift keying
SD	Secure digital
RAM	Random access memory
RPU	Separate processing unit
PCU	Single processing unit
HDD	Hard disk drive
